# Aphid-killing bacteria: diversity, origin, mechanisms and biocontrol potential

**DOI:** 10.3389/fmicb.2026.1805793

**Published:** 2026-03-31

**Authors:** Lara Berings, Nicolas Rojas-Preciado, Wouter Poppelsdorf, Sara Van Hee, Hans Jacquemyn, Bart Lievens

**Affiliations:** 1 CMPG Laboratory for Process Microbial Ecology and Bioinspirational Management (PME&BIM), Department of Microbial and Molecular Systems (M2S), KU Leuven, Leuven, Belgium; 2 Leuven Plant Institute (LPI), KU Leuven, Leuven, Belgium; 3 Laboratory of Plant Conservation and Population Biology, Department of Biology, KU Leuven, Leuven, Belgium

**Keywords:** aphids, biological control, entomopathogens, insect-microbe interactions, phyllosphere

## Abstract

Aphids are among the most destructive agricultural pests worldwide and cause substantial yield losses through direct feeding, virus transmission, and indirect plant damage. While chemical insecticides have been the primary control strategy of aphids, increasing resistance, environmental concerns, and regulatory restrictions have prompted the search for alternative approaches. In recent years, aphid-killing bacteria have emerged as a promising yet underexplored group of biological control agents. Growing evidence shows that aphids are susceptible to a diverse range of bacteria spanning multiple taxonomic groups. Many of these bacteria originate from plant-associated environments or from the aphids themselves and employ different mechanisms to reduce aphid survival and fitness, including toxin production, immune suppression, and disruption of aphid symbioses. This mini-review summarizes current knowledge on the diversity, ecological origins, and modes of action of aphid-killing bacteria. We further discuss their potential advantages, limitations, and challenges for practical implementation in biological control and integrated pest management strategies of aphids. Improved understanding of these bacteria may facilitate their application as effective, sustainable alternatives to chemical insecticides for aphid management.

## 1 Introduction

Aphids (Hemiptera: Aphididae) represent a highly diverse group of sap-feeding insects and are one of the most destructive agricultural pests worldwide (Kumar, 2020). Even at low densities, aphids can substantially reduce crop yields through direct feeding damage, induction of plant stress responses, and the transmission of plant viruses. Additionally, the secretion of aphid-produced honeydew promotes the growth of sooty molds, which reduces photosynthetic capacity and causes esthetic damage to fruits and vegetables (Dedryver et al., 2010). Direct and indirect damage by aphids is estimated to result in tens of millions to billions of euros in crop losses worldwide each year (Kim et al., 2008; Dedryver et al., 2010). In the UK alone, damage to cereals caused by aphids has been estimated at £60–120 million annually (Tatchell, 1989). Furthermore, their ability to reproduce parthenogenetically and to respond quickly to favorable environmental conditions leads to rapid population growth, making aphid outbreaks difficult to predict and control (Simon and Peccoud, 2018; Rojas-Preciado et al., 2026).

Over the past decades, aphid control has relied heavily on chemical insecticides. While initially effective, intensive insecticide use has led to widespread resistance development (Bass and Nauen, 2023). In addition, increasingly restrictive regulatory frameworks within the European Union have prompted a shift toward biological control and integrated pest management (IPM) approaches that aim to reduce chemical inputs while maintaining effective pest suppression. Microbial control agents represent a promising component in this transition, with entomopathogenic fungi and bacteria receiving particular attention due to their high specificity, environmental compatibility, and potential for persistence and self-propagation in agroecosystems (Lacey et al., 2015; Smee et al., 2021; Paliwal et al., 2022; Toledo-Hernánde et al., 2025). However, in contrast to their susceptibility to entomopathogenic fungi, which actively penetrate the cuticle and proliferate within their aphid hosts (Shah and Pell, 2003), aphids have long been considered relatively insensitive to bacterial entomopathogens. This is largely due to their piercing-sucking feeding mode, which limits ingestion of plant-applied microbial cells or toxins, and the historical focus on *Bacillus thuringiensis*, whose insecticidal cytolytic (Cyt) and crystal (Cry) *δ*-endotoxins show limited efficacy against hemipterans (Toledo-Hernánde et al., 2025).

More recently, this perception has changed substantially. A growing body of research indicates that aphids are susceptible to a surprisingly broad range of environmental bacteria spanning multiple taxonomic groups (Toledo-Hernánde et al., 2025). Many of these aphid-killing bacteria are not classical entomopathogens, which primarily thrive in soil (Gangwar et al., 2021), but instead originate from plant-associated environments such as the phyllosphere (Grenier et al., 2006; Walterson and Stavrinides, 2015; Smee et al., 2021; Paliwal et al., 2022) or from aphid-associated microbial communities (Harada and Ishikawa, 1997; Walterson and Stavrinides, 2015; Pons et al., 2019). These bacteria cause mortality within 1 to 3 days of topical application or ingestion or cause severe fitness reduction, through diverse modes of action, and have emerged as promising, yet underexplored, candidates for biological control. This mini-review summarizes current knowledge on the diversity and ecology of aphid-killing bacteria, their effects on aphid survival and fitness, their modes of action, and their potential applications and prospects in sustainable aphid management.

## 2 Diversity and ecology of aphid-killing bacteria

Aphid-killing bacteria encompass a broad range of taxonomic groups, including both Gram-positive and Gram-negative taxa. Reported taxa include members of the Bacillaceae (e.g., *Bacillus*) among Gram-positive bacteria, as well as Enterobacteriaceae (e.g., *Enterobacter*), Erwiniaceae (e.g., *Erwinia, Pantoea*), Pectobacteriaceae (e.g., *Dickeya*), Pseudomonadaceae (e.g., *Pseudomonas*), and a few other families among Gram-negative bacteria (reviewed in Toledo-Hernánde et al., 2025) (Table 1).

**Table 1:** Effects of aphid-killing bacteria in *in vitro* and *in planta* assays^a^.

Family	Species	Strain	Origin	Assay	Aphid species	Effect^b^ (%)	Mode of action	References
Bacillaceae	*Bacillus amyloliquefaciens*	CBMDDrag3 PGPBacCA2 CBMDLO3	Honey Honey Air	Oral feeding 10^8^ CFU/mL 4 days	*Myzus persicae*	100 100 100	NA	López-Isasmendi et al. (2019)
	*Bacillus pumilus*	PTB180	NA	Topical 10^8^ CFU/mL 7 days	*Aulacorthum solani* *Aphis gossypii*	38 50	NA	Kahia et al. (2021)
	*Bacillus subtilis*	PTB185	Plant tissue	Topical 10^8^ CFU/mL 7 days	*A. solani* *A. gossypii*	22 39	NA	Kahia et al. (2021)
Enterobacteriaceae	*Enterobacter xiangfangensis*	ER93	*Capsicum annuum*	Oral feeding 10^7^ CFU/mL 72 h	*M. persicae* *Aphis fabae* *A. solani* *Nasonovia ribisnigri* *Macrosiphum albifrons* *Brevicoryne brassicae*	63 27 67 40 20 53	NA	Paliwal et al. (2022)
Erwiniaceae	*Erwinia aphidicola*	IAM 14479 IAM 14480 IAM 14481 IAM 14482 IAM 14483	*Acyrthosiphon pisum* *A. pisum* *A. pisum* *A. pisum* *A. pisum*	Oral feeding 10^5^ CFU/mL 24 h	*A. pisum*	81 95 96 100 100	Gut infection Gut infection Gut infection Gut infection Gut infection	Harada and Ishikawa (1997)
	*Erwinia iniecta*	B120 B137	*Diuraphis noxia* *D. noxia*	Oral feeding 10^8^ CFU/mL 48 h	*D. noxia*	50 90	NA NA	Campillo et al. (2015)
	*Pantoea agglomerans*	PaR38	*Nasturtium officinale*	Oral feeding 10^7^ CFU/mL 72 h	*M. persicae* *A. fabae* *A. solani* *N. ribisnigri* *M. albifrons* *B. brassicae*	100 53 100 100 77 87	NA	Paliwal et al. (2022)
	*Pantoea stewartii*	DC283	NA	Oral feeding 10^8^ CFU/mL 72 h	*A. pisum*	NA^c^	Gut blockage	Stavrinides et al. (2010)
Pectobacteriaceae	*Dickeya dadanti* (syn. *Erwinia chrysanthemi*)	3937	NA	Oral feeding 10^7^ CFU/mL 4 days	*A. pisum*	100	Cyt-like toxin	Grenier et al. (2006)
Pseudomonadacea	*Pseudomonas fluorescens*	PpR24	*Brassica oleracea*	Oral feeding 10^7^ CFU/mL 72 h	*M. persicae* *A. fabae* *A. solani* *N. ribisnigri* *M. albifrons* *B. brassicae*	100 100 100 100 83 100	Tc proteins, Rhs elements, and a secreted protease (AprX)	Paliwal et al. (2022)
		PfR37	*Calendula officinalis*	Oral feeding 10^7^ CFU/mL 72 h	*M. persicae* *A. fabae* *A. solani* *N. ribisnigri* *M. albifrons* *B. brassicae*	100 100 73 57 93 90	NA	Paliwal et al. (2022)
	*Pseudomonas rhizosphaerae*	PrR91	*Foeniculum vulgare*	Oral feeding 10^7^ CFU/mL 72 h	*M. persicae* *A. fabae* *A. solani* *N. ribisnigri* *M. albifrons* *B. brassicae*	20 43 77 83 100 80	NA	Paliwal et al. (2022)
	*Pseudomonas syringae*	DSM50252	*Beta vulgaris*	Oral feeding 10^8^ CFU/mL 72 h	*M. persicae* *A. gossypii* *A. pisum* *Rhopalosiphum padi* *Aphis rumicis*	37–48^d^ 61–72^d^ 28–35^d^ 20–31^d^ 53–65^d^	NA	Smee et al. (2021)
		B728a	*Phaseolus vulgaris*	Oral feeding 10^8^ CFU/mL 72 h	*M. persicae* *A. gossypii* *A. pisum* *R. padi* *A. rumicis*	85–92^d^ 90–96^d^ 61–72^d^ 16–25^d^ 88–94^d^	NA	Smee et al. (2021)
		Cit7	Citrus	Oral feeding 10^8^ CFU/mL 72 h	*M. persicae* *A. gossypii* *A. pisum* *R. padi* *A. rumicis*	85–93^d^ 84–91^d^ 78–87^d^ 42–53^d^ 87–94^d^	NA	Smee et al. (2021)

Family	Species	Strain	Origin	Assay	Plant species	Aphid species	Effect (%)	Mode of action	References
Bacillaceae	*Bacillus pumilus*	PTB180	NA	Foliar spray 10^7^ CFU/mL 9 days	*Cucumis sativus* *Solanum lycopersicum*	*A. gossypii* *A. solani*	26^e^ 46^b^	NA	Kahia et al. (2021)
	*Bacillus subtilis*	PTB185	Plant tissue	Foliar spray 10^7^ CFU/mL 9 days	*Cucumis sativus* *Solanum lycopersicum*	*A. gossypii* *A. solani*	26^e^ 43^b^	NA	Kahia et al. (2021)
Pseudomonadaceae	*Pseudomonas fluorescens*	PpR24	*Brassica oleracea*	Foliar spray 10^7^ CFU/mL 21 days	*Beta vulgaris* *Capsicum annuum* *Arabidopsis thaliana*	*M. persicae* *M. persicae* *M. persicae*	69^e^ 68^e^ 57^e^	Tc proteins, Rhs elements, and a secreted protease (AprX)	Paliwal et al. (2022)

a NA: information not available.

b Mortality rate (%).

c Described as highly virulent; specific mortality rate (%) not reported.

d Range among three independent replicates.

e Population reduction (%) compared to control.

Several aphid-killing bacteria originate as epiphytic, plant-associated microbes, particularly from the phyllosphere. Many are also known plant pathogens or opportunistic phytopathogens, such as *Dickeya dadantii* (syn. *Erwinia chrysanthemi*) (Grenier et al., 2006), *Pantoea agglomerans* (Paliwal et al., 2022), *Pantoea stewartii* (Stavrinides et al., 2010) and *Pseudomonas syringae* (Stavrinides et al., 2009; Smee et al., 2021). Several phytopathogenic bacteria are thought to have initially exploited insects as vectors and, over time, evolved novel modes of interaction that, in some cases, result in the death of the insect hosts (Nadarasah and Stavrinides, 2011). Given the harsh conditions for survival and growth in the phyllosphere (e.g., nutrient limitation, fluctuating temperature and humidity, and high UV exposure), these phytopathogenic bacteria benefit from aphids as transient hosts that enable rapid multiplication and dispersal. During feeding, aphids acquire these bacteria from plant surfaces (Lou et al., 2025), after which they grow abundantly in the gut, and are subsequently excreted in honeydew as a concentrated inoculum onto leaves of the same or other plants (Stavrinides et al., 2009; Paliwal et al., 2022; Smee and Hendry, 2022). Once redeposited on leaves, the bacteria gain a competitive advantage over other phyllosphere microbes by efficiently exploiting honeydew-derived carbohydrates, promoting their growth and aphid-killing activity (Nadarasah and Stavrinides, 2011; Smee and Hendry, 2022). The bacteria are subsequently dispersed by wind, rain, or insect vectors, facilitating spread across plants and fields. Although it may seem counterintuitive that aphid-killing bacteria exploit aphids for growth and dispersal while also killing them, this apparent paradox can be interpreted through the classical virulence-transmission trade-off theory, which predicts that high virulence is favored when transmission occurs early and efficiently (Anderson and May, 1982; Ewald, 1983). In aphid-associated systems, rapid bacterial replication followed by excretion in honeydew enables bacterial dispersal prior to host death, while simultaneously enhancing pathogen fitness in the foliar environment, thereby compensating for the cost of killing the vector (Stavrinides et al., 2009; Smee and Hendry, 2022). Moreover, many aphid-killing bacteria maintain environmental reservoir phases on leaf surfaces, where they persist independently of insects (Hirano and Upper, 2000; Stavrinides et al., 2009). Such mixed transmission systems can buffer the fitness costs of high virulence, allowing aggressive exploitation of aphids while sustaining populations epiphytically between transmission events.

Other aphid-killing bacteria are primarily aphid-associated, including gut-associated taxa or endosymbiont-related lineages. The first bacteria described as pathogenic to aphids were *Erwinia aphidicola* strains isolated from the gut of healthy pea aphids (*Acyrthosiphon pisum*) (Harada et al., 1997). When administered via an artificial diet, the bacteria caused mortality in aseptically reared pea aphids lacking gut microbes. *E. aphidicola* proliferates in the aphid gut, potentially utilizing substrates like sucrose and trehalose, and its pathogenicity may be enhanced by high-density colonization, the absence of competing gut flora, or host stress turning a normally aphid-associated bacterium into an entomopathogen (Harada and Ishikawa, 1997). Although initially considered an aphid-associated bacterium, *E. aphidicola* has subsequently also been identified as a plant pathogen (Santos et al., 2009), reinforcing the fluid boundary between plant-associated and insect-associated lifestyles. Additionally, aphid-killing activity has been reported for bacteria that are primarily known as bacterial symbionts. For example, some culturable lineages of *Serratia symbiotica*, a facultative aphid endosymbiont known to enhance aphid tolerance to environmental stressors (Oliver et al., 2014), have shown to cause aphid mortality following oral exposure (Elston et al., 2021). *Serratia symbiotica* is phylogenetically closely related to *Serratia marcescens*, a bacterium that is widely recognized for its broad entomopathogenic activity (Akhtar et al., 2025). This indicates that relatively small shifts in host-microbe interactions can lead to aphid-killing phenotypes.

## 3 Effects on aphids and modes of action

Aphicidal activity varies substantially depending on the bacterial strain, aphid species, inoculum dose, and exposure route (Table 1). Aphids lack several genes that are crucial for defense against bacterial pathogens (Gerardo et al., 2010), making them highly susceptible to various bacterial groups. The strongest aphicidal effects are generally observed following ingestion rather than topical application. Ingestion enables bacterial colonization of the digestive tract and *in situ* toxin production, leading to mortality rates of up to 100% within 2 to 3 days in artificial diet feeding assays containing bacterial cell suspensions (Table 1). Although lethal effects have been observed at inoculum doses sometimes as low as 10 ingested bacterial cells (Grenier et al., 2006), the bacteria generally need to reach a threshold concentration of approximately 10^6^ cells per aphid to cause mortality (Stavrinides et al., 2009; Paliwal et al., 2022). Several aphid-killing bacteria are active against multiple aphid species, reflecting both the broad activity of bacterial virulence factors and the conserved gut physiology and immune responses across aphids (Smee et al., 2021; Ma and Lu, 2025). For example, *Pseudomonas fluorescens* strain PpR24 caused 100% mortality across multiple aphid species in *in vitro* feeding assays within 72 h of ingestion (Paliwal et al., 2022). Interestingly, this strain was also found effective against both insecticide-susceptible and insecticide-resistant aphid clones. When PpR24 was applied to leaves of *Arabidopsis thaliana*, sweet pepper (*Capsicum annuum*) and sugar beet (*Beta vulgaris*), *Myzus persicae* population sizes 21 days after bacterial inoculation and aphid infestation were reduced by 57, 68 and 69%, respectively (Paliwal et al., 2022). Similarly, epiphytic strains of *P. syringae* displayed broad aphicidal activity against diverse aphid species in artificial diet feeding assays (Stavrinides et al., 2009; Smee et al., 2017, 2021). Given this breadth of pathogenicity across aphid species, this suggests the possibility that aphid-killing bacteria may also infect other sap-sucking hemipterans. For example, the sweet potato whitefly (*Bemisia tabaci*) suffers mortality similar to that of pea aphids when exposed to certain *P. syringae* strains (Smee et al., 2017), but further research is needed to generalize this pattern.

**Figure 1:**
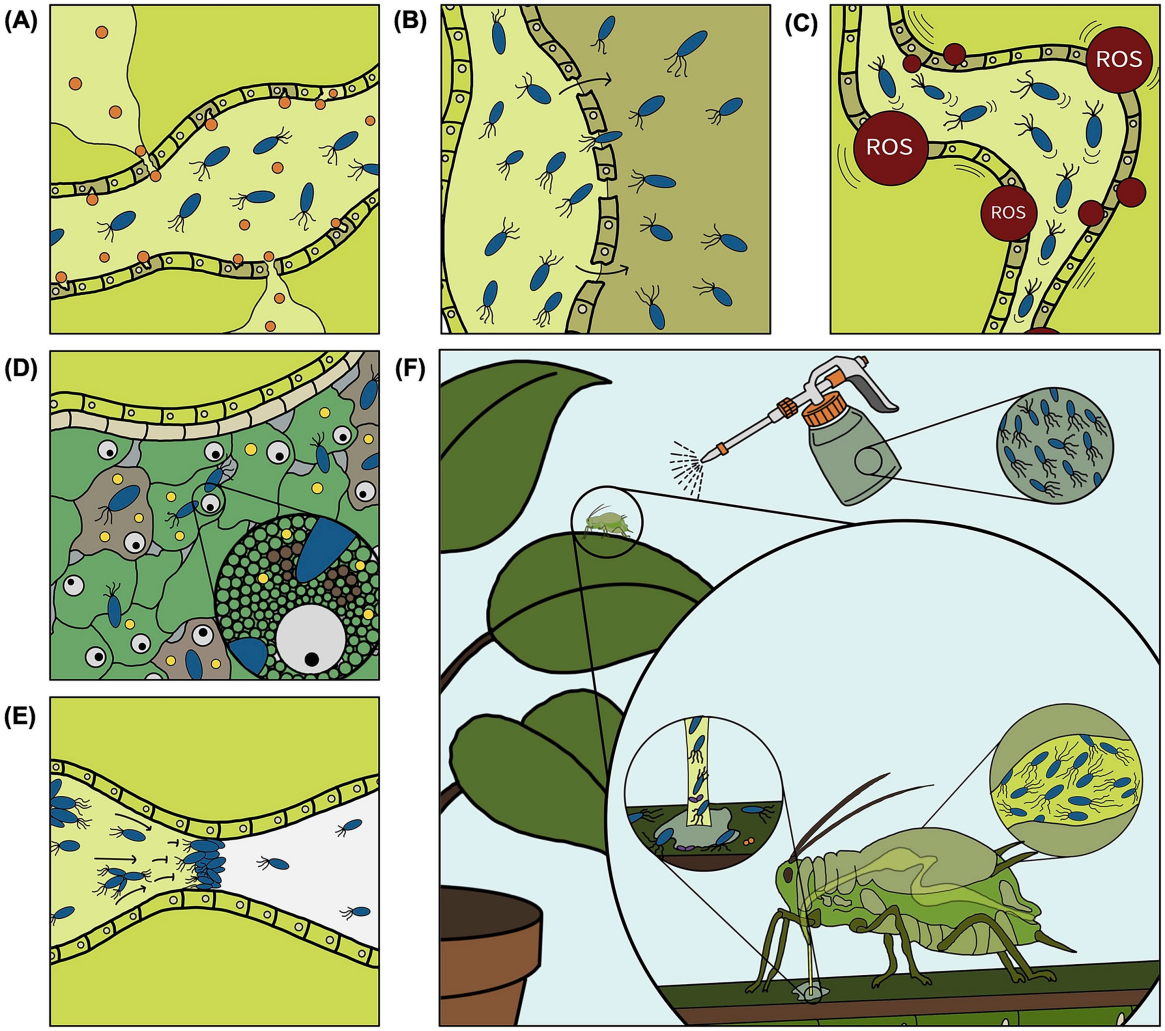
Schematic representation of aphid infection and mortality caused by aphid-killing bacteria. Following ingestion, bacterial pathogens kill the aphids through multiple mechanisms, including direct cytotoxicity due to toxin production (orange) **(A)**, gut colonization and septicemia **(B)**, metabolic collapse and oxidative stress, e.g., production of reactive oxygen species (ROS) **(C)**, symbiont-targeted disruption **(D)**, and physical obstruction **(E)**. These bacteria may provide novel strategies for aphid management **(F)**. Color code: light green indicates phloem sap in the aphid gut; olive green and gray indicate damaged cells or tissue.

Aphid-killing mechanisms can be categorized into five partially overlapping classes: (i) direct cytotoxicity, (ii) gut colonization and septicemia, (iii) metabolic collapse and oxidative stress, (iv) symbiont-targeted disruption, and (v) physical obstruction (Figures 1A–E).

(i) Direct cytotoxicity

Direct cytotoxicity (Figure 1A) involves bacterial toxins or metabolites that damage host cells through membrane disruption, pore formation, or enzymatic degradation. This mechanism is among the best-documented ways by which bacteria kill aphids, although only a limited number of aphicidal toxins or other secondary metabolites have been identified to date. A well-characterized example is *D. dadantii*, which produces Cyt-like pore-forming toxins in pea aphids that cause epithelial damage in the gut and facilitate bacterial invasion, leading to host mortality (Costechareyre et al., 2012). Similarly, in *P. fluorescens* PpR24, toxin complex (Tc) proteins, rearrangement hotspot (Rhs) elements and a secreted protease (AprX) contribute to aphicidal activity (Paliwal et al., 2024). In addition, biosurfactants such as dirhamnolipid and orfamide A produced by *Pseudomonas* spp. exhibit aphid-killing activity, likely through membrane-disruptive effects (Kim et al., 2011; Jang et al., 2013). These factors can be considered important virulence determinants as genetic deletion significantly reduces aphid mortality (Paliwal et al., 2024). However, deletion of such cytotoxic factors is often insufficient to abolish aphid mortality (Grenier et al., 2006; Paliwal et al., 2024), indicating that direct cytotoxicity is typically necessary but not sufficient for full virulence.

(ii) Gut colonization and septicemia

Following ingestion, successful colonization of the aphid gut can lead to septicemia, i.e., aphid death due to uncontrolled systemic bacterial proliferation in the hemolymph (Figure 1B). In *D. dadantii*, Cyt-like toxins are expressed specifically in the digestive tract, where they facilitate bacterial translocation into the hemocoel during the early stages of infection, ultimately causing death by septicemia (Costechareyre et al., 2012). Experimental comparisons between whole-cell treatments and cell-free filtrates further support the importance of active colonization, as concentrated culture supernatants often induce weaker or delayed mortality than live bacteria (López-Isasmendi et al., 2019; Paliwal et al., 2022).

(iii) Metabolic collapse and oxidative stress

Bacterial infection frequently induces the production of reactive oxygen species (ROS) in aphids, leading to oxidative stress, metabolic dysregulation, and immune challenges (Zhang and Lu, 2015) (Figure 1C). Transcriptomic analyses during PpR24 infection revealed altered expression of genes involved in detoxification, oxidative stress responses, and central metabolic pathways, consistent with systemic physiological disruption (Paliwal et al., 2024). Whether oxidative stress represents a primary virulence mechanism or a secondary consequence of infection is context-dependent. In some cases, ROS overproduction results from host immune activation, reflecting an opportunistic amplification of pathology. In other cases, bacterial factors may actively manipulate host redox homeostasis. Regardless of its origin, metabolic collapse often acts synergistically with direct cytotoxicity and septicemia and contributes to host death through multi-system failure (Li et al., 2023; Paliwal et al., 2024).

(iv) Symbiont-targeted disruption

Virtually all aphids rely on the obligate intracellular symbiont *Buchnera aphidicola* for essential amino acid provisioning absent from phloem sap (Douglas, 1998), as well as facultative symbionts like *Hamiltonella defensa*, *Regiella insecticola* and *Serratia symbiotica*, that modulate stress tolerance and protection against pathogens and natural enemies (parasitoids) (Oliver et al., 2010). For example, *H. defensa* can confer strong resistance to parasitoid wasps through toxin-encoding bacteriophages associated with the symbiont (Oliver et al., 2009), while *R. insecticola* has been shown to protect aphids against fungal pathogens such as *Pandora neoaphidis* (Scarborough et al., 2005) as well as certain viral pathogens (Higashi et al., 2023). Disruption of these associations can significantly impair host fitness and contribute to aphid mortality (Zhang et al., 2015) (Figure 1D). For example, a Cry41-related toxin from *B. thuringiensis* (Palma et al., 2014) was recently shown to impair *Buchnera* function by binding to its ATP-dependent 6-phosphofructokinase, resulting in aphid death (Jin et al., 2023).

(v) Physical obstruction

A distinct mechanism involves mechanical interference with gut function (Figure 1E). The transmembrane protein Ucp1 produced by *P. stewartii* promotes bacterial cell aggregation within the aphid gut, leading to physical blockage, impaired feeding, and eventual starvation and aphid death (Stavrinides et al., 2010). In this case, mortality results not from classical toxin-mediated cytolysis or metabolic disruption but from obstruction of nutrient flow.

## 4 Biocontrol potential and directions for further research

For decades, *B. thuringiensis* has been the most well-known and widely used bacterium for insect pest control (Sanchis and Bourguet, 2008; Azizoglu et al., 2023; Frentzel et al., 2026). However, although some experimental *B. thuringiensis* strains have shown aphicidal activity (Payne and Cannon, 1993), no aphid-killing *B. thuringiensis* strains have yet reached registration (Toledo-Hernánde et al., 2025). Nevertheless, the high mortality rates and reduced aphid population development observed *in vitro* and in small-scale controlled *in planta* assays (Table 1) indicate that entomopathogenic bacteria other than *B. thuringiensis* represent promising candidates for aphid biocontrol and integration into IPM strategies (Figure 1F). In addition, while these bacteria directly kill aphids, alternative microbial strategies could exploit symbionts such as *Wolbachia* that manipulate host reproduction, a phenomenon widely recognized for its potential in pest control (Zabalou et al., 2004; Werren et al., 2008). However, research supporting its application to aphid control remains scarce. Compared to chemical insecticides, aphid-killing bacteria may offer key advantages, including high host specificity, local persistence on plant surfaces, reduced environmental impact, and lower risk of resistance development (Table 2). They may also act more rapidly than natural enemies such as parasitoids, which often keep their hosts alive for extended periods of time (1–2 weeks) during which they can still feed on the plant (Boivin et al., 2012) (Table 2). However, strong selective pressures imposed by bacterial toxins may promote resistance development, as illustrated by the emergence of resistance to *B. thuringiensis* toxins in several lepidopteran pests under intensive use (McGaughey, 1985; Afzal et al., 2024). In addition, insect-associated microbiota can influence host susceptibility to entomopathogens (Zhang et al., 2024), which may enhance the capacity of aphids to withstand bacterial infections in the field. Furthermore, aphids may use visual cues, such as ultraviolet-based fluorescence produced by aphid-killing pseudomonads, to detect and avoid feeding on colonized leaves, thereby reducing their risk of infection (Hendry et al., 2018).

**Table 2:** Comparison of aphid-killing bacteria with common aphid control methods.

Parameter	Chemical pesticide	Parasitoids and predators	Aphid-killing bacteria
Time to aphid death	Immediate to short, depending on active ingredient	Immediate (predators) to delayed (koinobiont parasitoids: up to 1–2 weeks)	Relatively short (1–3 days) under favorable conditions, depending on bacterial strain, aphid species and inoculated dose
Effectiveness	High, rapid knockdown of aphid populations; can achieve near-total control	Moderate to high under favorable conditions and when natural enemy densities are sufficiently high; strongly dependent on environmental factors and availability of alternative prey	High under *in vitro* conditions, moderate under greenhouse or semi-field conditions, typically resulting in partial population reductions
Risk for resistance development	Very high; aphids frequently evolve resistance to active ingredients	Very low	Probably low; multifactorial modes of action reduce the risk for resistance development, but long-term field studies still limited
Specificity	Often broad-spectrum; can affect non-target insects including beneficials	High host specificity for parasitoids; predators may be more generalist	Generally high specificity toward aphids, but additional non-target evaluation needed
Cost	Variable; may be low initially, but repeated applications and resistance management increase total cost	Moderate; requires mass-rearing, regular releases to achieve sufficiently high densities, and/or habitat management	Moderate to high; formulation, storage, and field application can be expensive, but may reduce chemical input costs
Environmental impact	Can be high; environmental contamination, harm to beneficial insects, regulatory restrictions	Low; environmentally friendly, minimal non-target effects	Probably low; environmentally friendly, limited non-target effects, but further research needed
Persistence	Short-lived; repeated applications typically required	Can persist as long as natural enemy populations are maintained	Can persist for days to weeks on leaf surfaces depending on environmental conditions; formulation might increase their persistence
Ease of application	Easy; standard spraying equipment	Moderate; requires close monitoring, appropriate timing of releases and habitat management	Moderate; requires optimized formulations, doses, and delivery methods and timing

At present, data from greenhouse trials evaluating the efficacy of aphid-killing bacteria under practical conditions remain scarce. Curative application of *Bacillus pumilus* PTB180 and *Bacillus subtilis* PTB185 on greenhouse cucumber plants infested with melon aphid (*Aphis gossypii*), applied alone or in combination, reduced the number of live aphids 9 days after application by an average of 26% compared with the untreated control. Similarly, cumulative mortality of adults of the foxglove aphid (*Aulacorthum solani*) 9 days after application of the same bacterial treatments on greenhouse tomato plants was significantly higher than in the control, with mortality rates ranging from 43 to 46% (Kahia et al., 2021). Future research should focus on practical implementation and improving application strategies, including dose, delivery method and timing of application, ideally informed by knowledge of the bacteria’s mode of action, to maximize efficacy while minimizing costs and non-target effects. Aphid-killing bacteria can persist on leaf surfaces for several weeks at population densities of 10^5^–10^6^ CFU cm^−2^ (Paliwal et al., 2022), suggesting that they could be used not only curatively but also as a preventive measure. However, this possibility remains to be validated under field conditions. Suitable formulation strategies will be critical for successful implementation, as they can enhance bacterial shelf life, protect cells from desiccation and UV radiation, and improve adhesion and survival on leaf surfaces. The addition of nutrients tailored to these bacteria, such as specific carbon sources or amino acids, as well as surfactants, adhesives or protective carriers, can improve field persistence and efficacy (Leggett et al., 2011; Bejarano and Puopolo, 2020).

Beyond formulation and application optimization, bacterial performance may be further enhanced through genetic engineering or experimental evolution. Genetic engineering approaches, including recombinant DNA cloning, promoter-driven expression control, gene fusion to generate chimeric proteins, and chromosomal integration of toxin genes, have led to enhanced insecticidal activity, broader host range, and improved persistence on crops (Azizoglu et al., 2020). For example, enhanced expression of insecticidal crystal proteins in *B. thuringiensis* has been achieved through co-expression systems that increased toxin production and insect mortality in mosquito larvae (e.g., *Aedes albopictus*) compared to native strains (Akhtar et al., 2021). Such approaches have already been applied across multiple bacterial genera, including those that harbor aphid-killing members like *Bacillus* and *Pseudomonas*, demonstrating the potential for tailoring bacterial biocontrol agents (Azizoglu et al., 2020). However, despite these advantages, genetic modification remains time-consuming and subject to ongoing regulatory and societal debate. Experimental evolution offers an alternative approach to improve desired traits of microorganisms without resorting to genetic engineering (Manriquez et al., 2021), thereby circumventing regulatory restrictions associated with genetically modified organisms (GMOs). In this strategy, entomopathogenic bacteria are repeatedly passaged through an insect host, allowing selection across multiple infection cycles and resulting in enhanced host adaptation and increased virulence. This approach has been demonstrated with *B. thuringiensis* subsp. *kurstaki*, where strains repeatedly passaged through Cry1Ac-resistant *Plutella xylostella* larvae showed increased virulence (Dimitriu et al., 2023).

Importantly, aphid-killing bacteria should be compatible with other pest control strategies, including biological, cultural, and chemical approaches, to avoid antagonistic interactions and maximize overall efficacy. Their deployment should be designed to complement existing IPM programs, allowing these bacteria to function as part of a broader, sustainable pest control framework rather than as standalone agents. In this regard, although these bacteria are generally considered to exhibit relatively high host specificity, their potential impact on non-target arthropods and beneficial insects such as pollinators and natural enemies still remains to be investigated. Preliminary experiments exposing honeybee larvae or royal jelly to *P. fluorescens* PpR24 revealed no detectable adverse effects (Hamilton, 2015).

To date, phytotoxic effects of aphid-killing bacteria have not been reported. However, given that some aphid-killing bacteria belong to taxa that include well-known plant pathogens, such as *Dickeya*, *Pantoea* and *Pseudomonas syringae*, rigorous species- and strain-level characterization is essential to apply these bacteria safely as biocontrol agents. In these taxa, pathogenicity is often determined at the strain or pathovar level rather than the species level, reflecting differences in virulence gene repertoires and host range at intra-species level (Xin et al., 2018; Lorenzi et al., 2022). Furthermore, large-scale application of bacterial biocontrol agents may influence native phyllosphere microbial communities, potentially altering microbial network structure and microbial interactions (Zhang et al., 2008; Sylla et al., 2013). The leaf microbiome plays a key role in plant health, stress resistance and nutrient cycling, and its disruption could lead to unintended consequences for disease susceptibility or crop performance (Mahmoudi et al., 2024). Particular attention should be given to the possibility of horizontal gene transfer within leaf-associated microbial communities, including the mobility of toxin or virulence-associated genes, which are frequently located on plasmids or genomic islands in plant-associated bacteria (Smillie et al., 2010; Ghaly et al., 2024). Comprehensive risk assessment should therefore include evaluation of their potential impact on non-targets, phytotoxicity, microbiome perturbation, and gene transfer dynamics under relevant field conditions to ensure safe and efficient integration into IPM systems.

In conclusion, aphid-killing bacteria show considerable promise *in vitro* and in small-scale plant assays, but substantial challenges remain for their practical implementation. Key hurdles include achieving high and consistent field efficacy, e.g., through optimized application and formulation strategies, thoroughly assessing impacts on non-target organisms, and integrating these bacteria safely into existing pest management systems. Addressing these issues will be critical to fully realize their potential as sustainable and effective biocontrol agents.
